# IL-23, but not IL-12, plays a critical role in inflammation-mediated bone disorders

**DOI:** 10.7150/thno.41378

**Published:** 2020-03-04

**Authors:** Jiajia Xu, Jiao Li, Yiming Hu, Kerong Dai, Yaokai Gan, Jingyu Zhao, Mingjian Huang, Xiaoling Zhang

**Affiliations:** 1 Department of Orthopedic Surgery, Xinhua Hospital, School of Medicine, Shanghai Jiao Tong University, Shanghai 200092, China.; 2 The Key Laboratory of Stem Cell Biology, Shanghai Institutes for Biological Sciences (SIBS), Chinese Academy of Sciences (CAS); University of Chinese Academy of Sciences, Shanghai 200031, China.; 3 Shanghai Key Laboratory of Orthopaedic Implant, Department of Orthopaedics, Shanghai Ninth People's Hospital, Shanghai Jiao Tong University School of Medicine, Shanghai 200011, China.

**Keywords:** IL-12, IL-23, BMMSCs, bone formation/resorption, bone regeneration

## Abstract

Interleukin-12 (IL-12) and IL-23 are thought to have central roles in inflammation and are critical to pathologies associated with inflammation-induced bone disorders. The deletion of IL-12p40 (a common subunit of IL-12 and IL-23) can improve bone regeneration. However, the relative roles of IL-12 and IL-23 in bone disorders are largely unknown.

**Methods**: Ectopic bone formation and skull defect models were established to evaluate the relative roles of IL-12 and IL-23 in inflammatory bone disorders. Differences in bone mass among WT, IL-12p35^-/-^, and IL-12p40^-/-^ mice (young and elderly) were detected by micro-CT. Osteogenic and osteoclastic activities were explored using ELISA, qRT-PCR, and histological analysis. Moreover, the mechanisms by which IL-12 and IL-23 regulated the differentiation of BMMSCs and RAW264.7 cells were explored using Alizarin Red and tartrate-resistant acid phosphatase staining *in vitro*. Apilimod was used to inhibit IL-12 and IL-23 production *in vivo*.

**Results**: Mice deficient in IL-12p40 promoted bone formation and protected against aging-related bone loss. By contrast, bone loss was aggravated in IL-12^-/-^ mice, suggesting that IL-23 may play a dominant role in inflammation-related bone disorders. Mechanistically, IL-12 and IL-23 coupled osteogenesis and osteoclastic activities to regulate bone homeostasis and repair. IL-23 deficiency increased bone formation and inhibited bone resorption. Finally, apilimod treatment significantly improved bone regeneration and calvarial defect repair.

**Conclusion**: These data collectively uncover a previously unrecognized role of IL-23 in skeletal tissue engineering. Thus, IL-23 can act as a biomarker to predict diseases and treatment efficacy, and apilimod can be used as an effective therapeutic drug to combat inflammatory bone disorders

## Introduction

Bone remodeling and regeneration are complex and well-orchestrated processes that involve multiple cell types and signaling pathways, which can recover skeletal function 1. Bone marrow mesenchymal stem cells (BMMSCs) are an attractive cell source for tissue engineering and regenerative therapy 2. However, many factors can affect BMMSC-mediated bone repair and age-related bone loss. Bone defect 3,4 or aging 5 creates a proinflammatory environment with elevated levels of immune factors, which result in the failure of bone regeneration and turnover. Studies have focused on severe or prolonged inflammation 6, which leads to increased bone resorption and impaired bone formation 3,7. By contrast, rational regulation of inflammatory responses in time and concentration is critical to the final outcome of bone regeneration. Inflammatory cells (including neutrophils, monocyte/macrophages, and T cells) arrive to the fracture site in the first phase of wound healing 4,7,8. They remove the cell and extracellular matrix debris and secrete inflammatory and chemotactic mediators to recruit BMMSCs/osteoprogenitor cells from local niches 7. The mechanisms by which BMMSCs and inflammation interact during physiological and pathological processes to regulate bone turnover are still poorly understood. Therefore, defining important inflammatory factors and regulating them at appropriate levels can better help us expand our existing knowledge and provide effective strategies for inflammation-induced bone-related diseases.

Interleukin-12 (IL-12) and IL-23 play important roles in adaptive immunity 9. IL-12, which is composed of p35 and p40 subunits, is essential for the development of T helper (Th) 1 cells and promotes the secretion of interferon γ (IFN-γ). Meanwhile, IL-23 (consisting of p40 and p19 subunits) is a member of the IL-12 family and has a central role in IL-17-producing Th17 cell responses in inflammation. IL-12 and IL-23 contribute to inflammatory pathologies and numerous afflictions affecting humans 10. IL-12p40, a common subunit of IL-12 and IL-23, is critical to pathologies associated with bone disorders 6, osteoarthritis 11, 12, and rheumatoid arthritis 13, 14. Thus, ustekinumab, an IL-12p40 antagonist, has been approved to treat psoriatic arthritis and used in clinical testing for rheumatoid arthritis 15. We previously reported that IL-12 and IL-23 indirectly inhibited BMMSC osteogenesis and induced cell apoptosis via IFN-γ and IL-17 6. IL-12p40 depletion in C57BL/6J mice improved bone formation *in vivo*. However, the precise roles of IL-12 or IL-23 alone in bone healing and aging remain unclear. Many studies have been carried out to investigate the relative roles of IL-12 and IL-23 in inflammatory diseases. IL-23, rather than IL-12, plays a dominant role in rheumatoid arthritis 16,17 and central nervous system autoimmune inflammation 18. Mice deficient in IL-23 can be resistant to immune-mediated diseases. By contrast, IL-12p35 depletion (selectively lacking only IL-12) in mice showed no protection or accelerated disease in some models 15.

In this study, we found that IL-12 and IL-23 significantly impaired bone formation. To determine the relative roles of IL-12 and IL-23 in inflammation-mediated inhibition of bone regeneration and age-related bone loss, we used young and old gene-targeted mice lacking only IL-12 (p35^-/-^) or both (p40^-/-^). The data presented here indicate that the loss of IL-12 resulted in bone loss, whereas the absence of IL-23 increased bone mass and contributed to healing and tissue regeneration. Therefore, IL-23 is essential for inflammation-induced bone diseases. Furthermore, osteogenic and osteoclastic capacities were enhanced in IL-12p35^-/-^ mice. By contrast, IL-12p40^-/-^ mice showed an increase in bone formation but a decline in bone resorption. Mechanistically, IL-12 and IL-23 indirectly suppressed BMMSC osteogenesis by IFN-γ and IL-17. IL-12, IFN-γ, and IL-17 inhibited osteoclast differentiation and activity, whereas IL-23 was critical for induction of osteoclast formation. The inhibition of IL-12p40 by gene-targeted mice or apilimod (IL-12/23 antagonist) promoted bone formation *in vivo*. In addition, IL-12p40 deletion protected bone mass against aging-induced inflammation. Finally, our study suggests that IL-23 may play an important role in inflammation-mediated bone loss, and IL-12p40 exhibits potential as a target for the treatment of pathological bone diseases.

## Materials and Methods

### Mice

B6.129S1-*Il12a^tm1Jm^*/J (IL-12p35^-/-^) and B6.129S1-*Il12b^tm1Jm^*/J (IL-12p40^-/-^) mice were purchased from Jackson Laboratory (Bar Harbor, ME, USA). Male C57BL/6J and immunocompromised nude mice were purchased from the Shanghai Laboratory Animal Center of Chinese Academy of Sciences (Shanghai, China). Mice between 8 and 10 weeks of age were used. For all experiments, mice were randomized to control/knockout or vehicle/treatment group. Where applicable, mice were randomized to treatment with apilimod/IL-12/IL-23 or vehicle. The mice were housed in a specific pathogen-free animal facility of Shanghai Jiao Tong University School of Medicine. All procedures were approved by the Institutional Animal Care and Use Committee of the Shanghai Institutes for Biological Sciences of Chinese Academy of Sciences.

### Cells

Mesenchymal stem cells were isolated as described previously 19. Briefly, MSCs were isolated from the bone marrow of the tibia and femur of 8-week-old C57BL/6J mice. Single cells were cultured in Dulbecco's Modified Eagle Medium (DMEM) supplemented with 10% fetal bovine serum (FBS) and 1% penicillin-streptomycin (Hyclone, Logan, UT, USA) at 37 °C in the presence of 5% CO_2_. The non-adherent cells were removed by washing with phosphate-buffered saline (PBS) after 48 h, and the adherent cells were maintained with medium replenishment every 3 days. The cells were cultured and used at 5th passage. The murine macrophage cell line RAW264.7 (ATCC, Rockville, MD, USA) was maintained in DMEM supplemented with 10% FBS. The cells were tested for mycoplasma contamination every 3 months.

### Ectopic bone formation

C57BL/6J, nude mice, IL-12p35^-/-^ mice, or IL-12p40^-/-^ mice (*n*=4-5 per group, male, 8-10 weeks old) were randomly divided into different groups as detailed in Results. A total of 3.0 × 10^6^ BMMSCs was mixed with 45 mg of β-tricalcium phosphate (β-TCP) ceramic particles (Shanghai Bio-lu Biomaterials Co., Ltd., Shanghai, China) and subcutaneously implanted into the dorsal surface of mice. For *in vivo* evaluation of the effects of sustained IL-12 and IL-23 release, HyStem-HP Hydrogel (250 μL; ESI BIO stem cell solutions, Alameda, CA, USA) was covered on the surface of the implants containing 200 ng of IL-12 and 200 ng of IL-23 (Peprotech, Rocky Hill, New Jersey, USA). Apilimod (7 mg/kg body weight 20; MedChem Express, Monmouth Junction, NJ, USA) and vehicle were orally administered twice a week, starting three days before surgery, to determine the effect of IL-12/23 inhibitor on bone formation *in vivo*. The samples collected at 56 days were fixed in 4% paraformaldehyde (PFA) and decalcified with 12.5% ethylenediaminetetraacetic acid (EDTA; pH 7.0).

### Histological and immunohistochemical analysis

Femurs or calvarial bones of mice were scanned with Scanco Medical CT-40 instruments (Scanco Medical AG, Bruttisellen, Switzerland) at 8 μm resolution. After scanning, the samples were decalcified and processed using a standard method to produce paraffin sections. The decalcified tissues were stained with hematoxylin and eosin (H&E) solution. Quantification of the newly-formed mineralized tissue area was performed with ImageJ software (National Institutes of Health, USA). Alkaline phosphatase (ALP) staining was performed using the BCIP/NBT Alkaline Phosphatase Color Development Kit (Beyotime Institute of Biotechnology, Jiangsu, China) in accordance with the manufacturer's instructions. Tartrate-resistant acid phosphatase (TRAP) staining was performed with a TRAP kit (Sigma-Aldrich, St. Louis, MO, USA) to evaluate the morphology and localization of osteoclasts in the femur sections. Investigators performing microcomputed tomography (micro-CT) or histomorphometry analysis were blinded to the genotype and treatment group of each sample.

Immunohistochemistry was performed using standard protocols 6,21,22. The slides were incubated with primary antibodies anti-IL-12p40 (Abcam, Cambridge, MA, USA; ab106270, 1:150), anti-osteocalcin (Santa Cruz Biotechnology, Santa Cruz, CA, USA; sc-30045, 1:100), anti-ALP (Santa Cruz Biotechnology; sc-30203, 1:20), or anti-TRAP (Santa Cruz Biotechnology; sc-28204, 1:20). Horseradish peroxidase-conjugated secondary antibody (Maixin Biotech Co., Ltd, Fuzhou, China) was used to detect immunoreactivity. The slides were counterstained with Mayer's hematoxylin (Hongqiaolexiang Inc., Shanghai, China) and coverslipped using Permount mounting medium (Electron Microscopy Sciences, Hatfield, PA, USA). ImageJ was used to quantify the staining on IHC images.

### Real-time PCR (RT-PCR)

Calvarial bones were homogenized in TRIzol solution (Invitrogen, CA, USA). RNA was extracted according to the manufacturer's instructions. In brief, 1 µg of total RNA was converted into cDNA by using the RevertAid Reverse Transcriptase kit (Fermentas, UK). RT-PCR was carried using a ViiA 7 Real-Time PCR System (Life Technologies, Gaithersburg, MD, USA) for SYBR green-based quantification. The 2-ΔΔCT method was used to analyze gene expression 23. Experiments were performed in triplicate. The sequences of the primers are shown in **Supplementary Table 1**.

### ELISA

Mouse serum was collected from 2-month-old C57BL/6J mice, IL-12p35^-/-^ mice, or IL-12p40^-/-^ mice (*n*=6-8 per group). The levels of serum osteocalcin (OCN) and collagen type I cross-linked C-telopeptide (CTX-1) were quantified by the ELISA kits (Elabscience Biotechnology Co., Ltd., Wuhan, China) in accordance with the manufacturer's instructions.

### Osteogenic differentiation assay

For osteogenic differentiation, 100 nM dexamethasone, 50 μM ascorbic acid, and 10 mM β-glycerophosphate (Sigma-Aldrich) were added to confluent BMMSCs. Spleens were collected from C57BL/6J mice to elucidate the indirect mechanism of IL-12 and IL-23 on BMMSC osteogenesis. Single-cell suspensions were prepared by mechanical disruption in RPMI 1640 medium supplemented with 10% FBS and 1% penicillin-streptomycin (Hyclone). CD4^+^ T cells were isolated by magnetic sorting from splenocytes with a mouse CD4^+^ T cell isolation kit (Miltenyi Biotec, Bergisch Gladbach, Germany) and stimulated with 5 μg/mL anti-CD3 antibody and 2 μg/mL anti-CD28 antibody (eBioscience, San Diego, CA, USA). The cultures were supplemented with recombinant mouse IL-12 (10 ng/mL) and IL-23 (25 ng/mL). Supernatants were collected after 3 days of stimulation. BMMSCs were stimulated with the supernatants and rat IgG (0.3 µg/mL)/IFN-γ neutralizing antibody (0.3 µg/mL)/IL-17 neutralizing antibody (0.3 µg/mL) in osteogenic differentiation. The medium was changed every 3-4 days. After 14 days of osteogenic differentiation, the cells were fixed with 4% PFA for 15 min, washed three times with PBS, and stained with Alizarin Red solution (Sigma-Aldrich) for 30 min. For quantification, Alizarin Red precipitates were solubilized. The stained samples were incubated with 0.1 N sodium hydroxide and measured at 548 nm by using a Tecan Safire2 microplate reader (Tecan, Durham, NC, USA).

### Osteoclast differentiation assay

RAW264.7 (4.5 × 10^4^) cells were seeded into 48-well plate and differentiated into osteoclasts in the presence of the receptor activator of NF-κB ligand (RANKL, 75 ng/mL; Peprotech) with or without proinflammatory cytokines (IL-12, IL-23, IFN-γ, and IL-17 alone: 75 ng/mL; IL-12 + IL-23: 37.5 ng/mL each; IL-12 + IL-23 + IFN-γ + IL-17: 18.75 ng/mL each; Peprotech). The medium was changed every 2 days. After 3 days of osteoclast differentiation, the cells were fixed with 4% PFA and stained with a TRAP kit (Sigma-Aldrich).

### Proliferation assay

Cell proliferation was determined using Cell Counting Kit-8 (CCK-8; Beyotime Institute of Biotechnology). Briefly, 1 × 10^4^ RAW264.7 cells were seeded in a well of 96-well plate and stimulated with RANKL (75 ng/mL) and different cytokines (IL-12, IL-23, IFN-γ, and IL-17 alone: 75 ng/mL; IL-12 + IL-23: 37.5 ng/mL each; IL-12 + IL-23 + IFN-γ + IL-17: 18.75 ng/mL each). After 36 h, 20 μL of CCK-8 was added to each well and the cells were further incubated for 1 h. Absorbance at 450 nm was recorded using a Tecan Safire microplate reader (Tecan, Durham, NC, USA).

### Western blot

Western blot analysis was performed as previously described 19,24,25. Cells were lysed in RIPA buffer supplemented with PMSF and protease inhibitor cocktail (Roche, Basel, Switzerland) on ice. Protein samples were separated on 10% SDS-polyacrylamide gels and transferred to nitrocellulose membranes (GE Healthcare, Little Chalfont, Buckinghamshire, UK) by standard methods. The membranes were immunoblotted with primary antibodies overnight at 4 °C. Antibodies against the following were used: p-p65 (1:1000; Cell Signaling Technology, Danvers, MA, USA), p65 (1:1000; Cell Signaling Technology), MAPK Family Antibody Sampler Kit (1:1000; Cell Signaling Technology), phospho-MAPK Family Antibody Sampler Kit (1:1000; Cell Signaling Technology), and GAPDH (1:5000; Kangchen Bio-tech, Shanghai, China). Horseradish peroxidase (HRP)-conjugated antibodies were used and visualized with the chemiluminescence substrate (Merck Millipore, Billerica, MA, USA).

### *In vivo* calcein labeling

Calcein labeling was performed using a previously described method 6. Mice (*n*=5 per group) were injected intraperitoneally with calcein (10 mg/kg body weight; Sigma-Aldrich) at 10 and 3 days before sacrifice. Tibia and femur were fixed in 70% ethanol and embedded in LR white acrylic resin and sectioned. Pictures were taken by fluorescence microscopy (Carl Zeiss, Jena, Germany). Investigators were blinded to the genotype. Mineral apposition rate (MAR) is the distance between double labels divided by the time between labels.

### Calvarial bone defects

In brief, 1.0 mm calvarial defects were created in the parietal bones of mice (8 weeks old, *n* = 4-5 per group). Apilimod (7 mg/kg body weight; MedChem Express, Princeton, NJ, USA) was orally administered twice a week, starting 3 days before surgery, to inhibit the production of IL-12 and IL-23. The mice were euthanized at 4 weeks after the surgery. The parietal bones were harvested and fixed for 24 h in 4% PFA for further study. Investigators were blinded to the treatment group of each sample.

### Statistical analysis

Data were presented as mean ± SD. Statistical analysis was carried out by two-tailed Student's *t*-test or analysis of variance (ANOVA) using GraphPad Prism 7.0. A *P* < 0.05 was considered statistically significant.

## Results

### IL-23, rather than IL-12, is essential for the inflammation-mediated inhibition of bone regeneration

**Figure 1:**
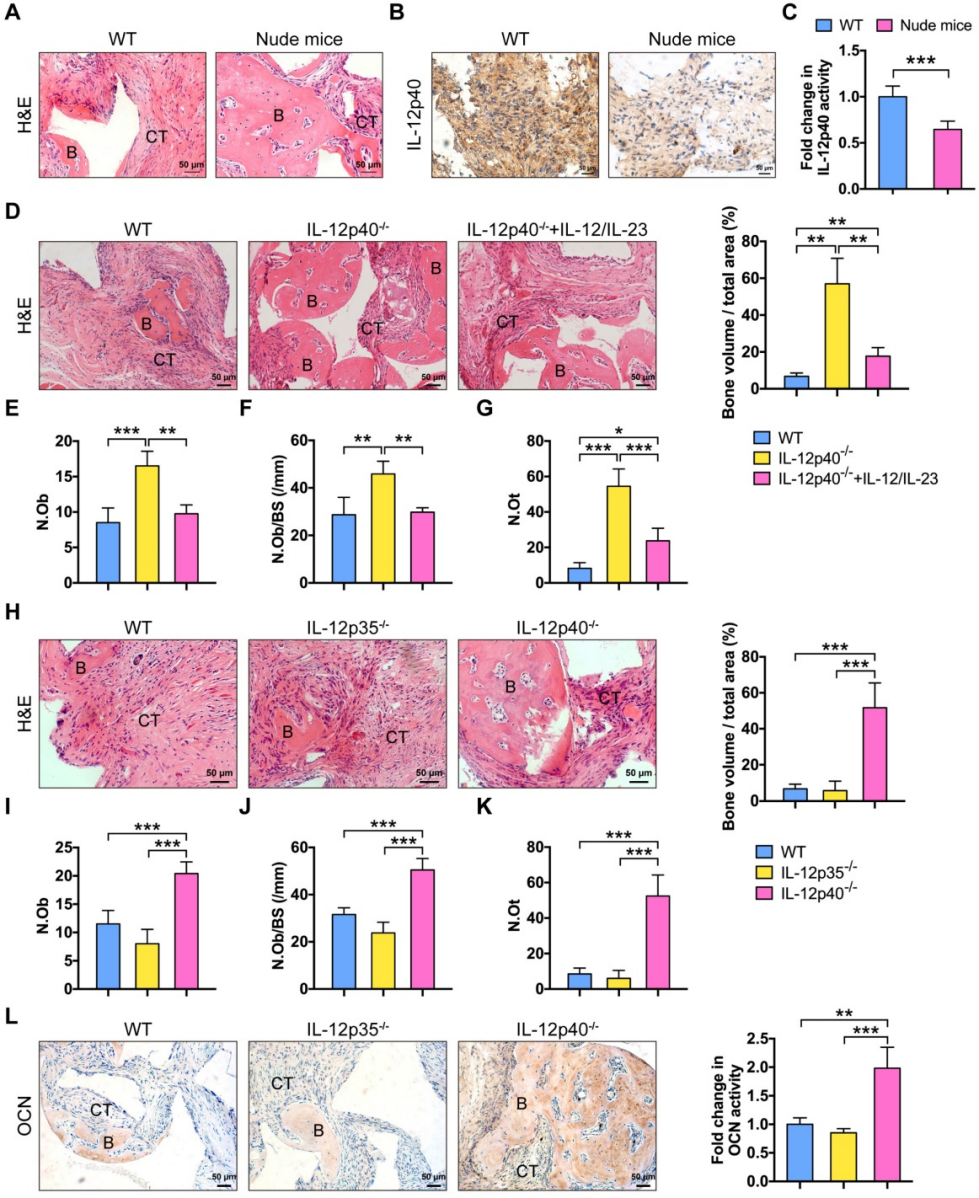
** IL-23 plays an essential role in inflammation-mediated inhibition of bone regeneration.** (**A**) BMMSCs mixed with β-TCP were implanted into the dorsal surface of WT and nude mice for 8 weeks. A substantial amount of bone was formed in nude mice, as detected by H&E staining. *n* = 4-5 per group. Scale bar, 50 µm. (**B**, **C**) IL-12p40 expression levels in implants after 7 days of implantation. Representative images (**B**) and quantification (**C**) of immunohistochemical staining of IL-12p40. *n* = 4-5 per group. Scale bar, 50 µm. (**D**) BMMSCs mixed with β-TCP plus IL-12 and IL-23 or no cytokine were implanted into the dorsal surface of WT and IL-12p40^-/-^ mice. New bone formation was detected with H&E staining. *n* = 4-5 per group. Scale bar, 50 µm. (**E-G**) Bone histomorphometric measurements among each group, including (**E**) osteoblast number (N.Ob), (**F**) osteoblast number per bone surface (N.Ob/BS), and (**G**) osteocyte number (N.Ot). (**H**) Representative images and quantification of ectopic bone formation in WT, IL-12p35^-/-^, and IL-12p40^-/-^ mice. *n* = 4-5 per group. Scale bar, 50 µm. (**I-K**) Bone histomorphometric measurements, including (**I**) N.Ob, (**J**) N.Ob/BS, and (**K**) N.Ot. (**L**) Osteocalcin (OCN) expression was increased in IL-12p40^-/-^ mice. Representative images and quantification of immunohistochemical staining of OCN were shown in WT, IL-12p35^-/-^, and IL-12p40^-/-^ mice. *n* = 4-5 per group. Scale bar, 50 µm. B, bone; CT, connective tissue; WT, wild-type. Results are shown as mean ± S.D. *p<0.05, **p<0.01, ***p<0.001.

BMMSCs are multipotent progenitor cells that can produce significant clinical improvements with bone repair 26. The biodegradable ceramics of β-TCP are widely used as bone regeneration materials 27. β-TCP combined with BMMSCs are transplanted into the site of defect to generate new bone and treat bone diseases. However, after the therapy, at least 5% of patients still proceed to delayed union or non-union 28. Inflammation impairs BMMSC-based tissue regeneration 6. T cells play a major role in this process 3. Nude mice lack T cells, resulting in an inhibited immune system. New bone formation was impaired in C57BL/6J mice but not in nude mice (**Figure 1A**). The amount of IL-12p40 (IL-12 and IL-23 share the p40 subunit 15) in the implants was significantly elevated in C57BL/6J mice (**Figure 1B-C**). IL-12 and IL-23 inhibited ectopic bone formation *in vivo*. The deletion of IL-12p40 promoted bone regeneration, demonstrating a significant increase in the numbers of osteoblasts and osteocytes [a 1.94-fold increase in the osteoblast number (Ob.N) and 1.60-fold increase when normalized to bone surfaces (Ob.N/BS) as well as 6.61-fold increase in the osteocyte number (N.Ot)]. By contrast, the addition of exogenous IL-12 and IL-23 efficiently blocked the new bone formation in IL-12p40^-/-^ mice, leading to 41% reduction in Ob.N, 35% reduction in Ob.N/BS, and 56% reduction in N.Ot (**Figure 1D-G**). These findings indicate that IL-12 and IL-23 play crucial roles in inhibiting bone regeneration *in vivo*.

To study the differential functions of IL-12 and IL-23 in bone formation, we compared new bone formation in the implants of mice lacking only IL-12 (IL-12p35^-/-^) or both cytokines (IL-12p40^-/-^). BMMSC-mediated bone formation was not increased in IL-12p35^-/-^ mice. Conversely, the implants showed a substantial amount of bone formation in IL-12p40^-/-^ mice, including a 1.65-fold increase in Ob.N, 1.46-fold increase in Ob.N/BS, and 6.27-fold increase in N.Ot in comparison with the wild-type (WT) control (**Figure 1H-K**). The immunostaining results of OCN in sections further supported the above observations. OCN expression was enhanced in animals lacking both IL-12 and IL-23 (IL-12p40^-/-^; **Figure 1L**). Thus, these data collectively suggest that inhibition of IL-23, but not IL-12, improves BMMSC-based bone regeneration, and the absence of IL-12 may reduce bone formation.

### Deletion of IL-23 promotes bone repair and increases bone mass

**Figure 2:**
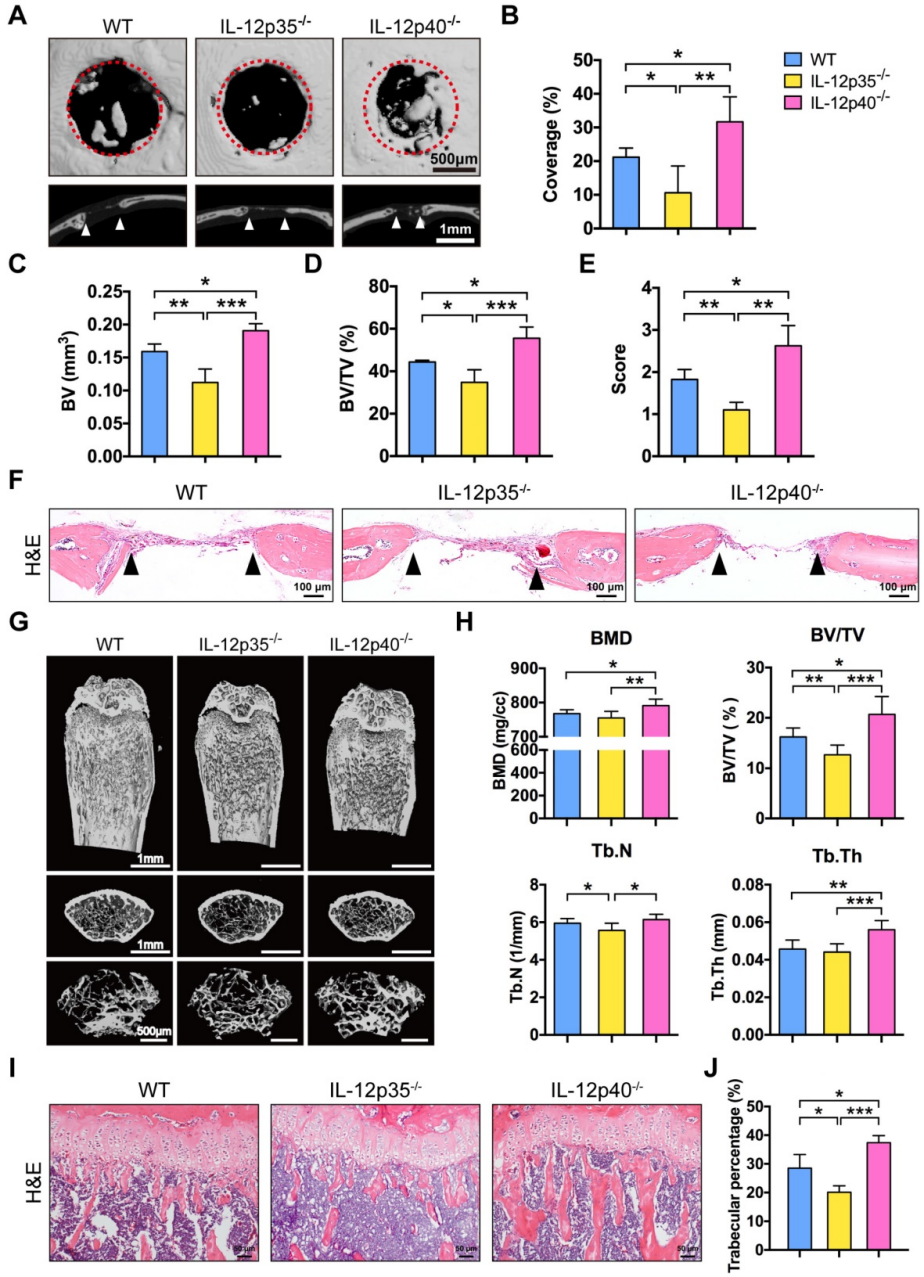
** IL-23 deficiency improves calvarial defect repair and enhances bone mass.** (**A**) Microcomputed tomography (micro-CT) three-dimensional reconstruction of bone repair in mouse calvarial defects. Red dotted lines indicate the periphery of the defect site. *n* = 4-5 per group. Scale bar, 500 µm (upper panel), 1 mm (lower panel). (**B**) Defect area coverage (%) was calculated via micro-CT images from WT, IL-12p35^-/-^, and IL-12p40^-/-^ mice. (**C-E**) Micro-CT analysis including (**C**) bone volume (BV), (**D**) bone volume/tissue volume (BV/TV), and (**E**) healing score for the extent of bony bridging and bone union. (**F**) The H&E staining of calvarial bone defects. *n* = 4-5 per group. Scale bar, 100 µm. (**G**) Representative images showed trabecular architecture by micro-CT three-dimensional reconstruction in distal femurs of 2-month-old mice. *n* = 4-5 per group. Scale bar, 1mm (upper and middle panel), 500 µm (lower panel). (**H**) Micro-CT measurements for the indicated parameters in distal femurs. Bone mineral density (BMD), BV/TV, trabecular numbers (Tb.N), and trabecular thickness (Tb.Th) were determined by micro-CT analysis. *n* = 4-5 per group. (**I**) The H&E staining of femur sections from 2-month-old WT, IL-12p35^-/-^, and IL-12p40^-/-^ mice were shown. *n* = 4-5 per group. Scale bar, 50 µm. (**J**) Trabecular percentage (%) was quantified via H&E images from the groups described in **I**. WT, wild-type. Results are shown as mean ± S.D. *p<0.05, **p<0.01, ***p<0.001.

We investigated whether IL-23 could affect bone repair and bone mass *in vivo*. In brief, 1.0 mm circular full thickness calvarial bone defects were created in WT mice, IL-12p35^-/-^, and IL-12p40^-/-^ mice. The defect re-ossification decreased in IL-12p35^-/-^ mice. However, large amounts of bone formation were detected in IL-12p40^-/-^ mice at 4 weeks after the surgery (**Figure 2A**). A semiquantitative analysis showed the percentage of new bone regeneration in each group (**Figure 2B**). The quantitative indices confirmed these findings across all metrics, including bone volume (BV, **Figure 2C**), bone volume/tissue volume (BV/TV, **Figure 2D**), and semi-quantitative healing score 29 (**Figure 2E**). The histomorphometric analysis showed that the defect gap was narrower in IL-12p40^-/-^ mice compared with that in WT and IL-12p35^-/-^ mice (**Figure 2F**). Micro-CT analysis of the distal femur metaphysis revealed that the bone mineral density (BMD) and BV/TV of IL-12p40^-/-^ mice were significantly higher than those of WT and IL-12p35^-/-^ mice at 2 months of age (**Figure 2G-H**). Similarly, the trabecular number (Tb.N) and trabecular thickness (Tb.Th) increased in IL-12p40^-/-^ mice (**Figure 2H**), consistent with the greater amount of trabecular bone shown in the H&E staining (**Figure 2I-J**). Thus, our findings suggest that loss of IL-23 in mice could enhance bone regeneration and bone mass.

### IL-12 and IL-23 regulate bone formation and bone resorption *in vivo*

**Figure 3:**
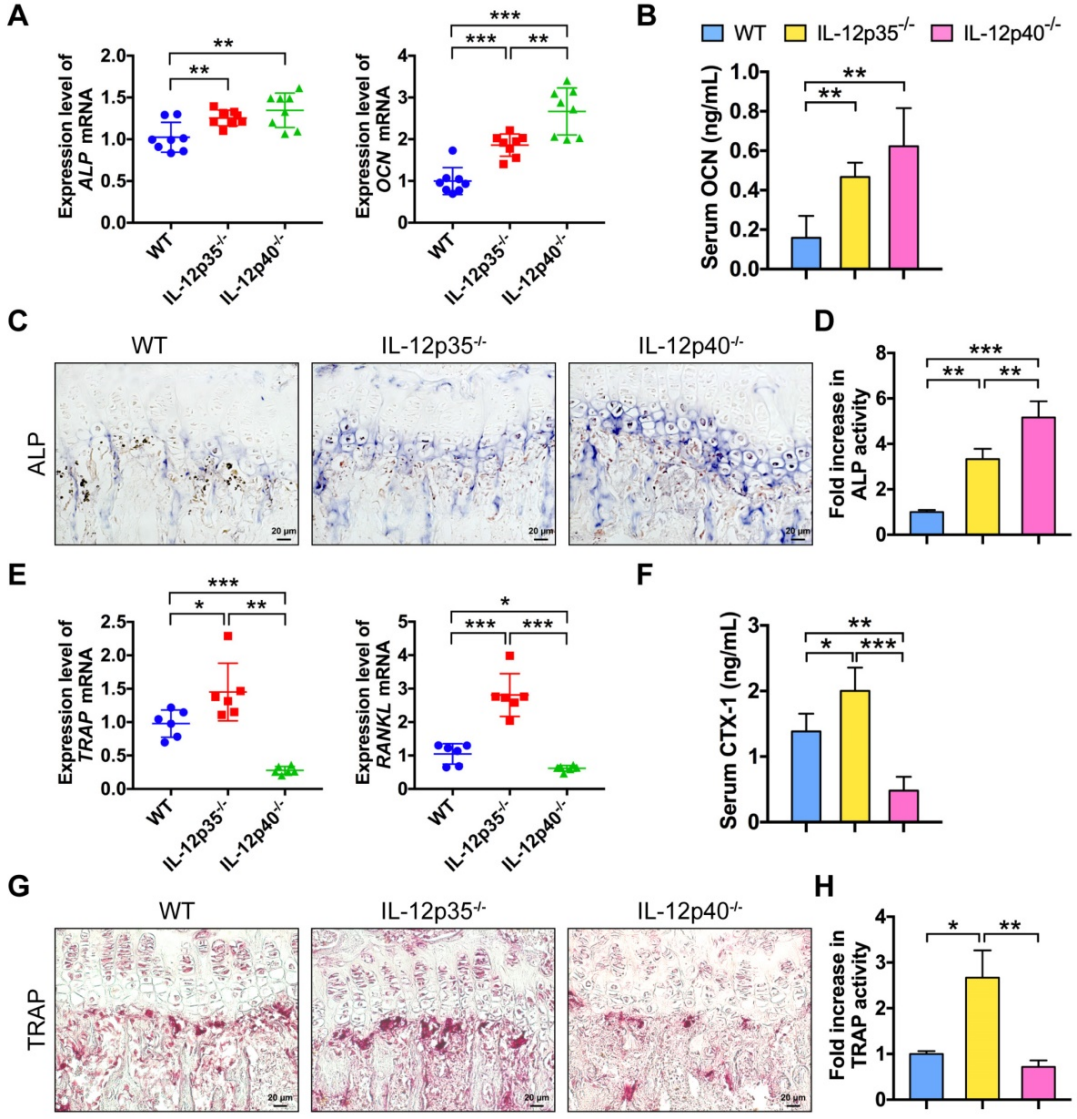
** IL-12 and IL-23 affect bone formation and resorption *in vivo*.** (**A**) The expression levels of osteoblast-related genes (*ALP* and *OCN*) in cranium from WT, IL-12p35^-/-^, and IL-12p40^-/-^ mice. *n* = 8 per group. (**B**) Serum levels of OCN. *n* = 8 per group. (**C**) ALP staining of femur sections from WT, IL-12p35^-/-^, and IL-12p40^-/-^ mice. *n* = 4-5 per group. Scale bar, 20 µm. (**D**) Quantification of ALP activity was shown via images from the groups described in **C**. (**E**) The expression levels of osteoclast-specific genes (*TRAP* and *RANKL*) in calvarial bone from WT, IL-12p35^-/-^, and IL-12p40^-/-^ mice. *n* = 6 per group. (**F**) Serum levels of CTX-1. *n* = 6 per group. (**G**) TRAP staining of femur sections from WT, IL-12p35^-/-^, and IL-12p40^-/-^ mice. *n* = 4**-**5 per group. Scale bar, 20 µm. (**H**) Quantification of TRAP activity was shown via images from the groups described in **G**. ALP, alkaline phosphatase; CTX-1, collagen type I cross-linked C-telopeptide; OCN, osteocalcin; RANKL, receptor activator of NF-κB ligand; TRAP, tartrate-resistant acid phosphatase; WT, wild-type. Results are shown as mean ± S.D. *p<0.05, **p<0.01, ***p<0.001.

To further understand how IL-12 and IL-23 regulate bone mass, we examined bone formation and resorption in mice. We compared gene expression profiles in bone among WT, IL-12p35^-/-^, and IL-12p40^-/-^ mice. The mRNAs of genes critical for osteogenesis, such as ALP and OCN, were largely elevated in IL-12p35^-/-^ and IL-12p40^-/-^ mice (**Figure 3A**). Furthermore, the serum levels of OCN, a marker for bone formation, were upregulated by 293% in IL-12p40^-/-^ mice, which was significantly greater than the 195% increase observed in IL-12p35^-/-^ mice (**Figure 3B**). The dynamic histomorphometry results further confirmed that the mineral apposition rate was significantly increased in IL-12p35^-/-^ (65% increase) and IL-12p40^-/-^ (108% increase) mice compared with that in WT mice (**Figure S1A-B**). Consistent with the enhanced bone formation in knockout mice, ALP staining revealed more bone-forming surface in IL-12p35^-/-^ mice than that in the WT mice. Moreover, the activities of ALP were stronger in IL-12p40^-/-^ mice than in IL-12p35^-/-^ mice (**Figure 3C-D**). Similar results were obtained by ALP immunohistochemistry (**Figure S1C**). IL-12 depletion led to markedly increased expression of TRAP and RANKL, while IL-12 and IL-23 depletion reduced the expression (**Figure 3E**). In addition, the serum levels of CTX-1, a bone resorption marker, were markedly increased in IL-12p35^-/-^ mice but decreased in IL-12p40^-/-^ mice (**Figure 3F**). We then evaluated whether the changed bone resorption is caused by osteoclastogenesis in the knockout mice. The bone histomorphological examinations showed that the number of TRAP^+^ osteoclasts was significantly elevated in the femurs of IL-12p35^-/-^ mice and reduced in the femurs of IL-12p40^-/-^ mice, as evidenced by the TRAP staining and immunohistochemistry results (**Figure 3G-H and Figure S1D-E**). Taken together, IL-12 depletion enhances bone formation and bone resorption. However, the depletion of both IL-12 and IL-23 promotes osteogenesis and inhibits osteoclastogenesis *in vivo*.

### IL-12 and IL-23 inhibit osteogenesis by IFN-γ and IL-17

**Figure 4:**
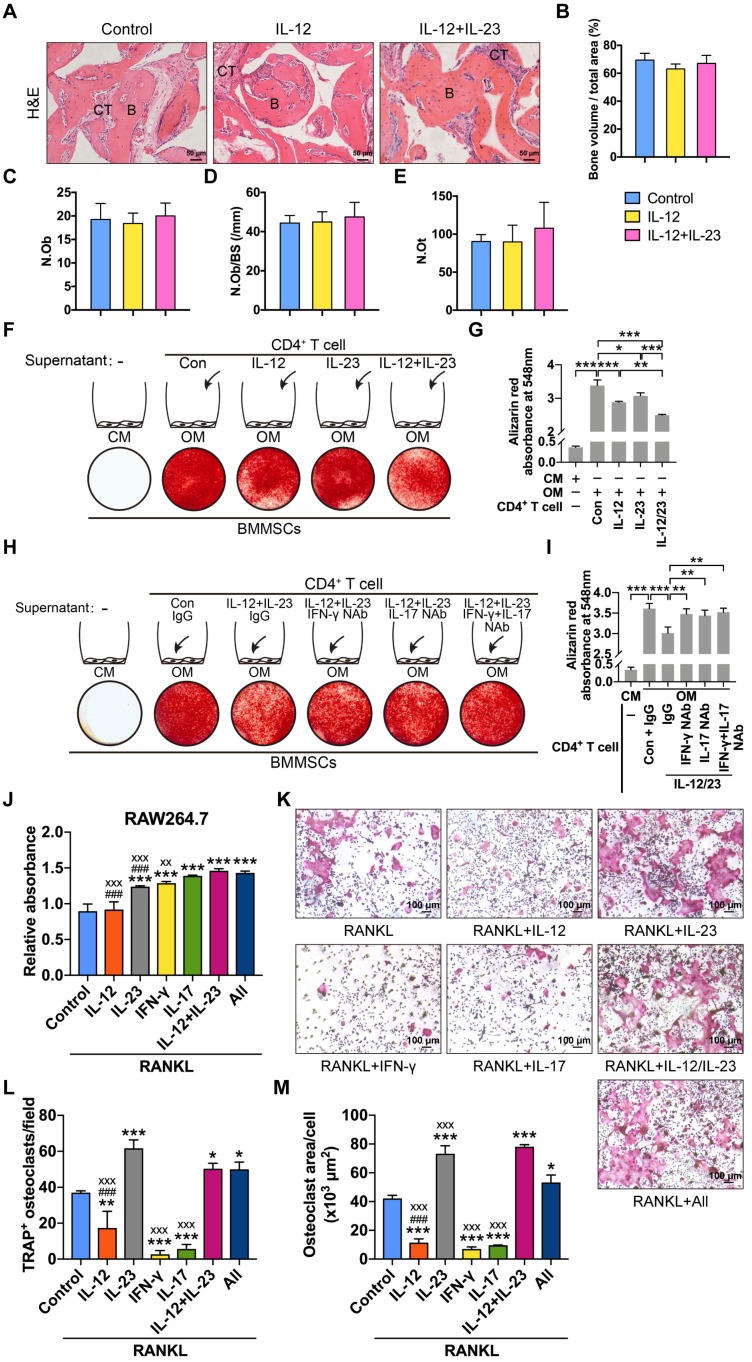
** IL-12 and IL-23 regulate BMMSC osteogenesis and osteoclast differentiation.** (**A**,** B**) BMMSCs mixed with β-TCP plus IL-12 or IL-12+IL-23 were subcutaneously implanted into immunocompromised mice for 8 weeks. (**A**) New bone formation was detected with H&E staining. *n* = 4-5 per group. Scale bar, 50 µm. (**B**) Quantitative analysis of new bone volume. *n* = 4-5 per group. (**C-E**) Bone histomorphometric measurements among each group, including (**C**) osteoblast number (N.Ob), (**D**) osteoblast number per bone surface (N.Ob/BS), and (**E**) osteocyte number (N.Ot). (**F**) Compared to untreated CD4^+^ T cell culture supernatants, IL-12- or/and IL-23-stimulated supernatants showed reduced capacities to form mineralized nodules of BMMSCs, as assessed by Alizarin Red staining. (**G**) Quantitative analysis of the calcium mineralization in **F**. (**H**) The blockade of IFN-γ or/and IL-17 in IL-12- and IL-23-stimulated supernatants rescued reduced capacities to form mineralized nodules in BMMSCs. (**I**) Quantitative analysis of the calcium mineralization in **H**. (**J**) RAW264.7 cell proliferation analysis treated with RANKL (75 ng/mL) and different cytokines. All: IL-12 + IL-23 + IFN-γ + IL-17. *: compared with control group; #: compared with IL-12 + IL-23 group; x: compared with All group. (**K**) Effects of different cytokines (IL-12, IL-23, IFN-γ, and IL-17) on the RANKL-induced osteoclast differentiation of RAW264.7 cells. (**L, M**) The number of TRAP^+^ osteoclasts per field (**L**) and the average size of osteoclasts (**M**). All: IL-12 + IL-23 + IFN-γ + IL-17. *: compared with control group; #: compared with IL-12 + IL-23 group; x: compared with All group. B, bone; CT, connective tissue; Con, control; CM, control medium; IFN-γ, interferon γ; OM, osteogenic medium; RANKL, receptor activator of NF-κB ligand. Results are shown as mean ± S.D. *p<0.05, **p<0.01, ***p<0.001; ^###^*p*<0.001; ^xx^*p*<0.01; ^xxx^*p*<0.001.

We explored the roles of IL-12 and IL-23 in BMMSC-mediated bone formation and found that IL-12 or IL-23 treatment did not affect the new bone formation in nude mice (**Figure 4A-E**). IL-12 and IL-23 regulate the differentiation of T helper cells 15. The supernatants of IL-12- or IL-23-stimulated T cells inhibited BMMSC osteogenesis (**Figure 4F-G**). Moreover, the combination of IL-12 and IL-23 displayed stronger suppression of matrix mineralization compared with that of either cytokine alone (**Figure 4F-G**). Taken together, these results collectively suggest that IL-12/IL-23 indirectly inhibit osteoblast differentiation via CD4^+^ T cells.

IL-12 and IL-23 increase the expression of IFN-γ and IL-17 in CD4^+^ T cells 6. We speculated that IL-12 and IL-23 impair osteogenic differentiation of BMMSCs by IFN-γ and IL-17. To confirm this hypothesis, we treated BMMSCs with IFN-γ/IL-17 neutralizing antibodies. The culture supernatants of T cells stimulated with IL-12 plus IL-23 significantly reduced mineralized nodule formation. Conversely, the blockage of IFN-γ and IL-17 alone or in combination rescued BMMSC osteogenesis, as assessed by Alizarin Red staining (**Figure 4H-I**). Therefore, IL-12 and IL-23 indirectly suppress the osteogenic potential of BMMSCs by promoting T cells to produce IFN-γ and IL-17.

### IL-12 and IL-23 regulate osteoclastogenesis by direct and indirect mechanisms

IL-12 and IL-23 induced the production of IFN-γ and IL-17 15, respectively. To explore the effects of IL-12 and IL-23 on RANKL-induced osteoclastogenesis, we incubated RANKL-treated RAW264.7 cells with IL-12, IL-23, IFN-γ, or IL-17. IL-12 treatment did not affect cell proliferation but reduced the number of TRAP^+^ osteoclasts (**Figure 4J-M**). IL-23 induced the proliferation of RAW264.7 cells, promoted osteoclast formation, and increased the average cell surface area compared with the control. Moreover, a combination of IL-12 and IL-23 also promoted cell proliferation and rescued the inhibitory effect by IL-12 on osteoclast formation. IFN-γ or IL-17 increased the proliferation rate, but significantly inhibited RANKL-induced osteoclastogenesis. Finally, a combination of IL-12, IL-23, IFN-γ, and IL-17 enhanced proliferation and osteoclast differentiation of RAW264.7 cells (**Figure 4J-M**). These results indicate that IL-12 and IL-23 influence the formation of functional osteoclasts. IL-23 can overcome the inhibitory effects of IL-12, IFN-γ, and IL-17 on osteoclastogenesis.

### Deletion of IL-23 attenuates aging-mediated bone loss

**Figure 5:**
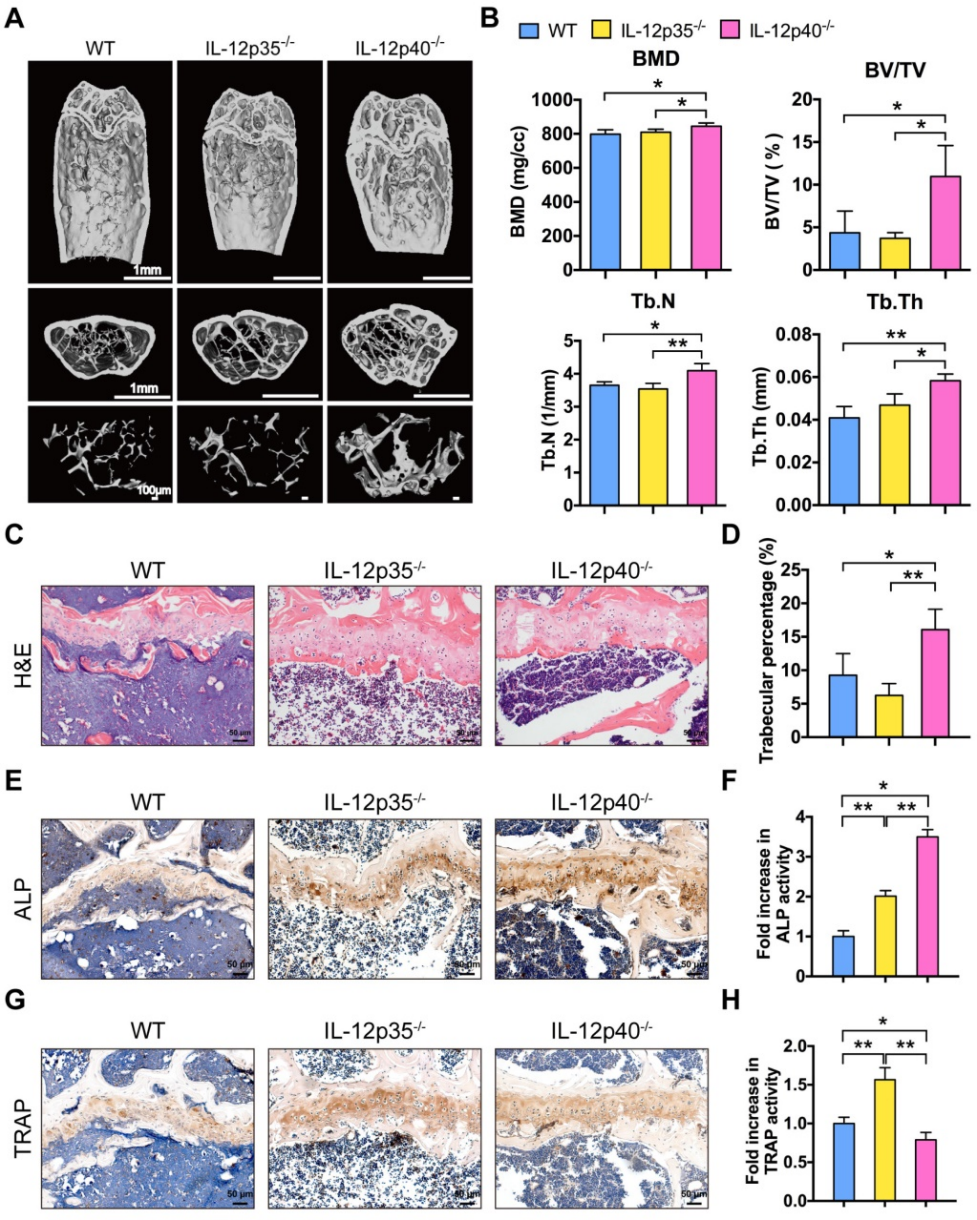
** IL-23 plays a critical role in aging-mediated bone loss.** (**A**) Micro-CT images of the trabecular bone in the distal femoral metaphysis of 12-month-old male WT mice and age-sex matched IL-12p35^-/-^ and IL-12p40^-/-^ mice. *n* = 4 per group. Scale bar, 1mm (upper and middle panel), 100 µm (lower panel). (**B**) Micro-CT measurements for the indicated parameters in distal femurs. Bone mineral density (BMD), bone volume/tissue volume (BV/TV), trabecular numbers (Tb.N), and trabecular thickness (Tb.Th) were determined by micro-CT analysis. *n* = 4 per group. (**C**) The H&E staining of femur sections from 12-month-old WT, IL-12p35^-/-^, and IL-12p40^-/-^ mice were shown. *n* = 4 per group. Scale bar, 50 µm. (**D**) Trabecular percentage (%) was quantified via H&E images from the groups described in **C**. *n* = 4 per group. (**E**, **F**) The immunohistochemical analysis (**E**) and quantification (**F**) of ALP expression in the distal femoral metaphysis. *n* = 4 per group. Scale bar, 50 µm. (**G**, **H**) The immunohistochemical analysis (**G**) and quantification (**H**) of TRAP expression in the distal femoral metaphysis. *n* = 4 per group. Scale bar, 50 µm. ALP, alkaline phosphatase; TRAP, tartrate-resistant acid phosphatase; WT, wild-type. Results are shown as mean ± S.D. *p<0.05, **p<0.01.

Aging is associated with impaired bone regeneration 30. Osteoporosis is a relevant age-related disorder. Although osteoporosis is not traditionally considered an immune disease, recent data have indicated that a proinflammatory environment is created in aging mouse models that leads to osteoporosis 5,31,32. The levels of IL-12p40 in elderly subjects were increased compared with the younger subjects 33. We further examined whether the inhibition of IL-12 or IL-23 could protect against aging-related bone loss and osteoporosis. Consistent with young adult mice, WT and IL-12p35^-/-^ mice showed comparable bone mass at 12 months of age. However, the trabecular bone mass markedly increased in 12-month-old IL-12p40^-/-^ mice (**Figure 5A**). Meanwhile, IL-12p40^-/-^ mice had greater BMD, BV/TV, Tb.N, and Tb.Th than age-matched WT and IL-12p35^-/-^ mice (**Figure 5B**). The histologic staining also revealed a trend of increased trabecular bone mass in aged IL-12p40^-/-^ mice (**Figure 5C-D**). The immunohistochemical staining of the bone sections demonstrated the high expression of ALP and TRAP in aged IL-12p35^-/-^ mice, resulting in a slight decrease in bone mass. However, aged IL-12p40^-/-^ mice had significantly increased bone mass with higher ALP activity and reduced TRAP activity (**Figure 5E-H**). These results thus suggest that inhibition of IL-23 ameliorates aging-related osteoporosis and promotes bone mass.

### Apilimod treatment improves BMMSC-mediated bone regeneration

**Figure 6:**
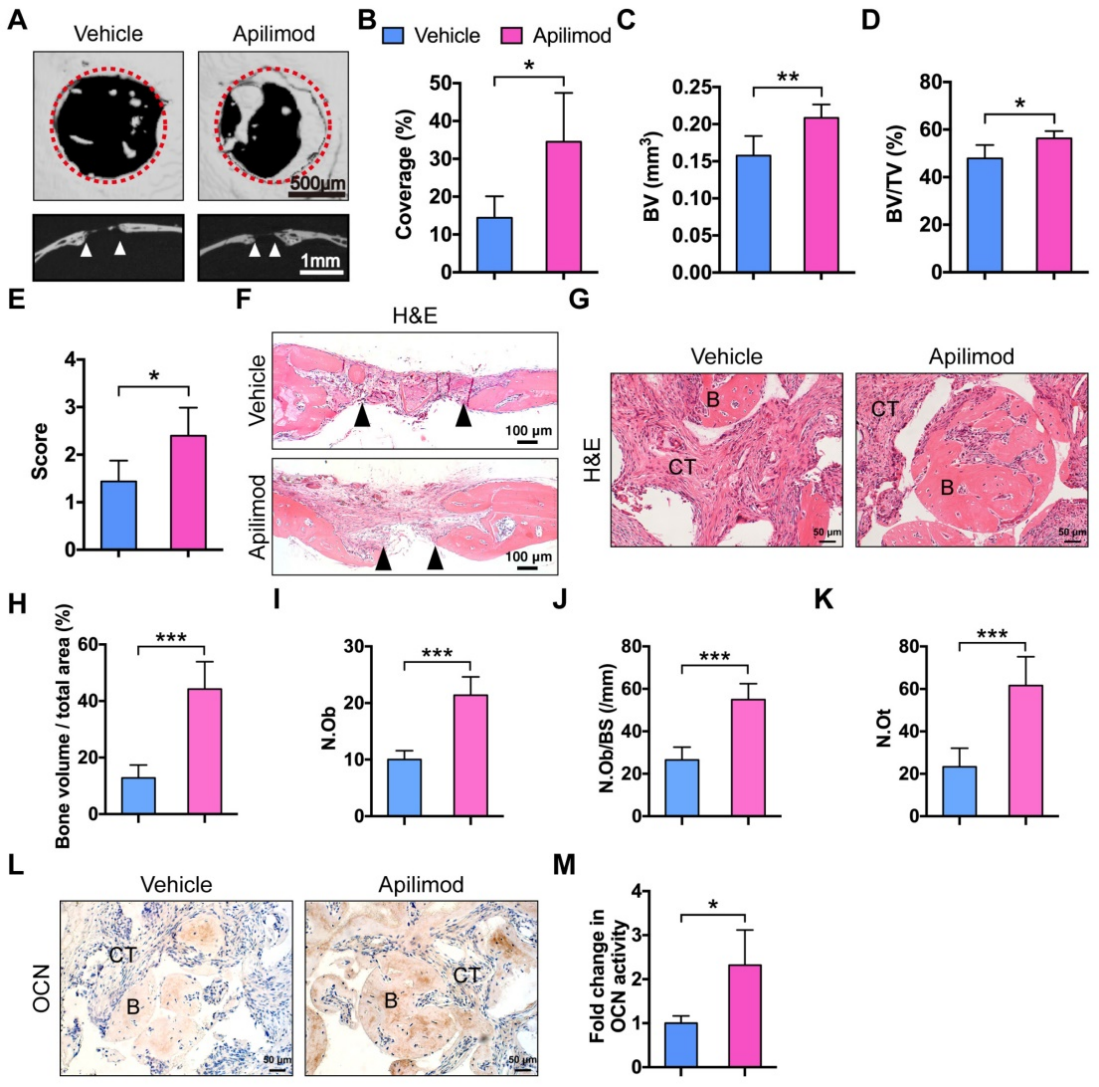
** Apilimod treatment improves calvarial defect repair and BMMSC-mediated bone formation.** (**A**) Micro-CT three-dimensional reconstruction of calvarial bone repair in WT mice after treatment with vehicle or apilimod. Red dotted lines indicate the periphery of the defect site. *n* = 5 per group. Scale bar, 500 µm (upper panel), 1 mm (lower panel). (**B**) Defect area coverage (%) was calculated via micro-CT images. *n* = 5 per group. (**C-E**) Micro-CT analysis including (**C**) bone volume (BV), (**D**) bone volume/tissue volume (BV/TV), and (**E**) healing score for the extent of bony bridging and bone union. *n* = 5 per group. (**F**) The H&E staining of calvarial bone defects. Scale bar, 100 µm. (**G**, **H**) BMMSCs mixed with β-TCP were implanted into the dorsal surface of WT mice for 8 weeks. The mice were given vehicle or apilimod. (**G**) New bone formation was detected with H&E staining. *n* = 5 per group. Scale bar, 50 µm. (**H**) Quantitative analysis of new bone volume. *n* = 5 per group. (**I-K**) Bone histomorphometric measurements among each group, including (**I**) osteoblast number (N.Ob), (**J**) osteoblast number per bone surface (N.Ob/BS), and (**K**) osteocyte number (N.Ot). (**L**, **M**) Representative images (**L**) and quantification (**M**) of immunohistochemical staining of OCN in implants. *n* = 5 per group. Scale bar, 50 µm. B, bone; CT, connective tissue; OCN, osteocalcin; WT, wild-type. Results are shown as mean ± S.D. *p<0.05, **p<0.01, ***p<0.001.

Apilimod was used as an inhibitor of IL-12 and IL-23 cytokine production and an immunomodulatory agent for treatment of inflammatory diseases in clinical trials 34,35. We investigated whether inhibition of IL-12/23 by apilimod could improve bone defect repair and BMMSC-based bone regeneration. We first generated subcritical-sized calvarial bone defects in WT mice and orally administered apilimod twice a week for 4 weeks. We then performed micor-CT analysis. Mice treated with apilimod showed significantly promoted bone formation and increased bone volume and semi-quantitative healing score (**Figure 6A-E**). The H&E staining results further supported the bone regeneration facilitated by IL-12p40 inhibition (**Figure 6F**). We further investigated the effect of apilimod on the bone formation model. We subcutaneously implanted BMMSCs and β-TCP in WT mice that were administered apilimod twice a week for 8 weeks. The apilimod treatment significantly improved bone formation compared with the vehicle group (**Figure 6G-H**), presenting a 2.14-fold increase in Ob.N, 2.07-fold increase in Ob.N/BS, and 2.66-fold increase in N.Ot (**Figure 6I-K**). The immunohistochemical staining results revealed intensive OCN in the apilimod-treated group (**Figure 6L-M**). Taken together, our results suggest that IL-12p40 inhibition by apilimod is a potential and ideal therapeutic approach for inflammation-mediated inhibition of bone regeneration and age-related bone loss.

## Discussion

The inflammatory response during ectopic bone formation in WT mice was more severe than that in nude mice, resulting in bone regeneration failure 3,7. However, the specific mechanism is still not entirely clear. We found that the IL-12p40 levels were elevated in WT mice. Although the role of IL-12p40 in bone formation is well established 6, the relative contributions of IL-12 and IL-23 to inflammation-induced bone loss and pathologies remain unclear. We used IL-12p35^-/-^ and IL-12p40^-/-^ mice to detect the effects of IL-12 and IL-23 on bone mass maintenance, ectopic bone formation, and calvarial bone defect healing. IL-12 knockout led to weakened osteogenic capacity and aggravation of bone loss, while the combined deletion of IL-12 and IL-23 promoted bone regeneration and increased bone mass. These results suggest that inhibition of IL-12 does not protect against inflammation-induced bone loss but rather has a more adverse effect. Taken together, IL-12 does not serve as an effective therapeutic target. Compared with IL-12 deletion, IL-23 knockout could effectively protect bone mass and improve bone repair. This finding further demonstrates that IL-23 is an essential regulator of inflammation-induced pathological bone diseases.

IL-12 and IL-23 have no direct effect on osteogenic differentiation of BMMSCs 6,36. IL-12 is primarily produced by macrophages and dendritic cells and promotes the secretion of IFN-γ 15,37,38, which induces cell apoptosis and suppresses BMMSC osteogenesis 6. Moreover, both IL-12 and IFN-γ are potent inhibitors of osteoclast differentiation 39-41. IL‐12 regulates NFATc1 expression and inhibits the osteoclastogenic activity of RANKL in bone marrow-derived monocytes (BMMs) and RAW264.7 cells 42. In addition, IL-12 enhances tumor necrosis factor (TNF)-α-induced osteoclast apoptosis by Fas/Fas ligand interaction 43. IFN-γ is a major product of activated Th cells 44 and directly blunts osteoclastogenesis through suppression of cathepsin K levels 45 or degradation of tumor necrosis factor receptor-associated factor 6 46,47 *in vitro*. Therefore, compared with WT mice, IL-12p35^-/-^ mice exhibit enhanced osteogenic and osteoclastogenic abilities, while the osteoclast activity is stronger *in vivo*. This phenomenon eventually leads to bone loss in IL-12p35^-/-^ mice. IL-23 overexpression in mice causes extensive bone loss and elevates IL-17 levels 48. IL-23-deficient mice show higher trend in bone mass 48. This result also confirms our hypothesis that targeting IL-23 can be an effective means for treatment of inflammation-induced bone diseases. IL-23 induces the production of IL-17 49-51. IL-17 inhibits the osteogenic differentiation of BMMSCs via the NF-κB and MAPK signaling pathways, and stimulation with IFN-γ further enhances cell apoptosis and inhibitory effects on osteoblast mineralization 3,6,52. Overall, the osteogenesis ability is stronger in IL-12p40^-/-^ mice than in IL-12p35^-/-^ mice. IL-23 strongly promotes osteoclast formation by direct upregulation of RANK in precursor cells 53 and indirect activation of RANKL expression on CD4^+^ T cells 54. Furthermore, osteoclast activity and resorbing capacity are impaired in BMMs isolated from IL-23^-/-^ mice 48. IL-17 decreases the production of cathepsin K and matrix metalloproteinase-9 and suppresses the differentiation of osteoclast precursors into osteoclasts 55. IL-17 impaired osteoclast differentiation by activating NF-κB, p38, and Erk signaling pathways in RAW264.7 cells (**Figure S2**). A combination of IL-12, IL-23, IFN-γ, and IL-17 promoted osteoclast differentiation, suggesting that the osteoclast-inducing ability of IL-23 should be significantly stronger than that of IL-12, IFN-γ, and IL-17. This finding may partly explain the weakening of osteoclastic ability after IL-12p40 knockout. However, deletion of IL-12 and IL-23 affects the development of Th1 and Th17 cells. IFN-γ and IL-17 are the major cytokines secreted by Th1 and Th17 cells. Except for these inflammatory factors, other cytokines may be involved in this process 56. Therefore, further research is needed to clarify the specific mechanism. IL-23 knockout promotes bone formation and suppresses osteoclastogenesis. Hence, IL-23 plays a more important role in bone repair and bone reconstruction.

Aging can cause chronic inflammation in elderly subjects 30. Recent evidence suggests that inflammation is also a cause of senile osteoporosis 32,56. The levels of proinflammatory cytokines were elevated in aged individuals, including IL-1, TNF-α, and IL-12p40 33,57. Since aging creates a proinflammatory microenvironment, we examined the roles of IL-12 and IL-23 in age-related bone loss. In this study, we found that IL-23 plays an important role in senile osteoporosis, and the absence of IL-23 can effectively inhibit bone loss. Moreover, the loss of IL-12 does not reverse bone mass. This finding provides a new theoretical basis for the pathogenesis of osteoporosis and can provide a reference for improving and accelerating therapeutic drug development.

Inhibition of IL-12p40 production can significantly reduce disease progression and promote tissue regeneration 16,18. Ustekinumab (an IL-12p40 neutralizing antibody) has been approved for treatment of psoriatic arthritis 15. The use of the IL-12p40 antagonist is subjected to other preclinical trials (Crohn's disease, rheumatoid arthritis, and multiple sclerosis), which also demonstrate the potential of IL-12p40 as a therapeutic target. However, IL-23 plays a more essential role than IL-12. IL-23 is involved in many bone diseases associated with inflammation, including nonunion 6, rheumatoid arthritis 58,59, and psoriatic arthritis 60,61. IL-23p19 antagonist (Guselkumab) has also been used for treatment of rheumatoid arthritis and psoriatic arthritis 15. In summary, we demonstrate that IL-23 is a previously unrecognized regulator of bone reconstruction and regeneration. Targeting IL-12p40 can promote tissue repair and protect bone mass, whereas IL-12 deletion paradoxically mediates bone loss.

## Supplementary Material

Supplementary figures and table.

## Abbreviations

ALP: alkaline phosphatase
ANOVA: analysis of variance
BMD: bone mineral density
BMMs: bone marrow-derived monocytes
BMMSC: bone marrow mesenchymal stem cell
β-TCP: β-tricalcium phosphate
BV: bone volume
BV/TV: bone volume/tissue volume
CCK-8: cell counting kit-8
DMEM: dulbecco's modified eagle medium
EDTA: ethylenediaminetetraacetic acid
FBS: fetal bovine serum
H&E: hematoxylin and eosin
HRP: horseradish peroxidase
IFN-γ: interferon γ
IL: interleukin
MAR: mineral apposition rate
micro-CT: microcomputed tomography
N.Ot: osteocyte number
Ob.N: osteoblast number
Ob.N/BS: osteoblast number/bone surfaces
OCN: osteocalcin
PBS: phosphate-buffered saline
PFA: paraformaldehyde
RANKL: receptor activator of NF-κB ligand
RT-PCR: real-time PCR
Tb.N: trabecular number
Tb.Th: trabecular thickness
Th: T helper
TNF: tumor necrosis factor
TRAP: tartrate-resistant acid phosphatase
WT: wild-type
