# Supplementary material for: IL-23, but not IL-12, plays a critical role in inflammation-mediated bone disorders
Source: Theranostics. 2020 Mar 4;10(9):3925–38. doi: 10.7150/thno.41378 (PMC7086346; doi:10.7150/thno.41378)
Supplement: Supplementary file 1 — Supplementary figures and table. [file thnov10p3925s1.pdf]

Supplementary Figure

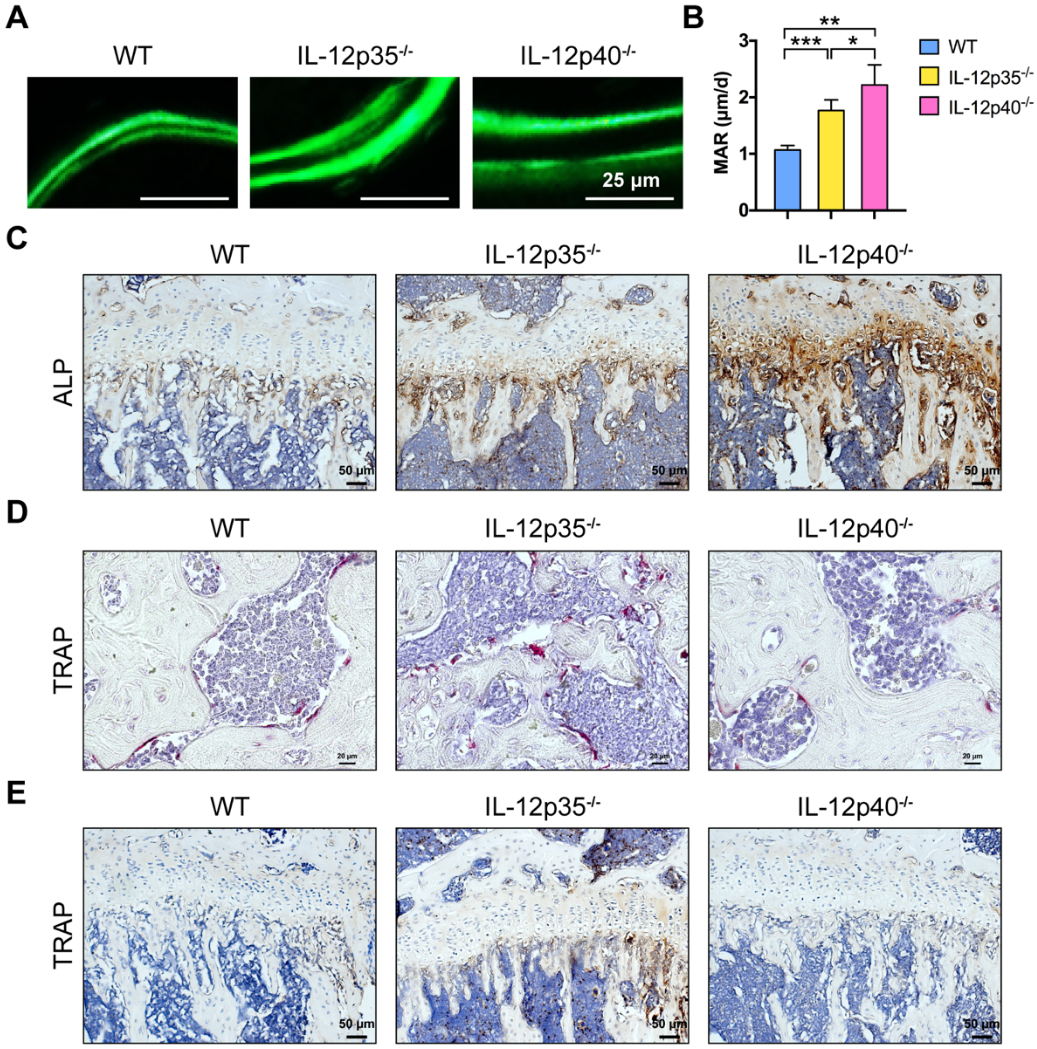

Figure S1. Coupling the activities of bone formation and resorption by IL-12 and IL-23.

(**A**) Representative images of new bone formation at distal femur metaphysis assessed by calcein double labeling in 2-month-old WT, IL-12p35$^{-/-}$, and IL-12p40$^{-/-}$ mice. n = 5 per group. Scale bar, 25 μm. (**B**) Quantification of bone histomorphometric results, indicated by mineral apposition rate (MAR). (**C–E**) Bone formation and resorption were detected by ALP and TRAP activity, respectively. (**C**) The immunohistochemical staining for ALP on sections of mouse femurs. n = 5 per group. Scale bar, 50 μm. (**D**) TRAP staining of distal femur sections from WT, IL-12p35$^{-/-}$, and IL-12p40$^{-/-}$ mice. n = 5 per group. Scale bar, 20 μm. (**E**) The immunohistochemical staining for TRAP on sections of mouse femurs. n = 5 per group. Scale bar, 50 μm. ALP, alkaline phosphatase;

TRAP, tartrate-resistant acid phosphatase; WT, wild-type. Results are shown as mean ± S.D. *p<0.05, **p<0.01, ***p<0.001.

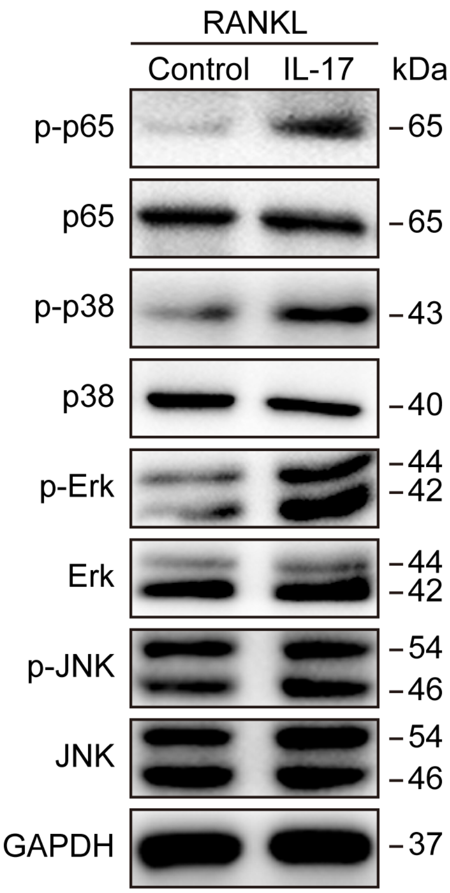

Figure S2. IL-17 activates NF-κB and MAPK signaling pathways in RAW264.7 cells.

Western blot analysis showed that IL-17 (75 ng/mL) impaired osteoclast differentiation by activation of NF-κB, p38, and Erk signaling pathways. RANKL, receptor activator of NF-κB ligand.

Supplementary Table 1: Primers used for quantitative real-time PCR.

Genes	Forward primer	Reverse primer
ALP	AGGGCAATGAGGTCACATCC	GCATCTCGTTATCCGAGTACCAG
OCN	AAGCAGGAGGGCAATAAGGT	TTTGTAGGCGGTCTTCAAGC
TRAP	ACACAGTGATGCTGTGTGGCAACTC	CCAGAGGCTTCCACATATATGATGG
RANKL	CAGCATCGCTCTGTTCCTGTA	CTGCGTTTTCATGGAGTCTCA
β-actin	ACTGGGACGACATGGAGAAG	GGGGTGTTGAAGGTCTCAAA

ALP: alkaline phosphatase; OCN: osteocalcin; TRAP: tartrate-resistant acid phosphatase; RANKL: receptor activator of NF-κB ligand.
